# Roles and usages of neuro service dogs for caregivers living at home with persons with dementia: An exploratory comparative case study

**DOI:** 10.1177/14713012231204646

**Published:** 2023-10-10

**Authors:** Claude Vincent, Frédéric S Dumont, Annette Rivard, Manon Rogers, Suzette Brémault-Phillips, Cary Brown, Bertrand Achou

**Affiliations:** School of Rehabilitation Sciences, 4440 Université Laval, Canada; Centre of Interdisciplinary Research in Rehabilitation and Social Integration (CIRRIS), 560498 Centre Intégré Universitaire de Santé et de Services Sociaux de la Capitale-Nationale (CIUSSS/CN), Canada; Centre of Interdisciplinary Research in Rehabilitation and Social Integration (CIRRIS), 560498 Centre Intégré Universitaire de Santé et de Services Sociaux de la Capitale-Nationale (CIUSSS/CN), Canada; Department of Occupational Therapy, Faculty of Rehabilitation Medicine, 3158 University of Alberta, Canada; Centre of Interdisciplinary Research in Rehabilitation and Social Integration (CIRRIS), 560498 Centre Intégré Universitaire de Santé et de Services Sociaux de La Capitale-Nationale (CIUSSS/CN), Canada; Department of Occupational Therapy, Faculty of Rehabilitation Medicine, 3158 University of Alberta, Canada; Centre of Interdisciplinary Research in Rehabilitation and Social Integration (CIRRIS), 560498 Centre Intégré Universitaire de Santé et de Services Sociaux de la Capitale-Nationale (CIUSSS/CN), Canada; Faculty of Economics and Business · Economics, Econometrics & Finance — Management. University of Groningen, Netherlands.

**Keywords:** service dog, assistance dog, caregivers, home care support, community dwelling, independence, socialisation

## Abstract

The goal of the present study was to examine how canine assistance may support family caregivers and persons with dementia and to document and compare two modalities of home care support. An exploratory comparative case study research design was conducted. Three cases correspond of dyads of a caregiver, a person with mild to moderate dementia, and either a neuro service dog (NSD), a companion dog or no dog. Hypotheses are formulated to capture differences between cases. Recruitment was done in a service dog organisation, through Canadian Alzheimer associations and in records of a hospital. Data were collected through 45–60 minutes telephone interviews that included completion of the Caregiver’s Burden Scale and sociodemographic questions. We used an inductive approach with qualitative data. There were five caregivers (mean age 54.8 years) who had an NSD, 28 caregivers (63.6 years) who had a companion dog, and 23 caregivers (63.8 years) without dog. In the category of roles and usages of the dog, ‘Socialisation’ and ‘Help with a sense of direction’ were the most addressed roles for dyads with the NSD. For dyads with companion dog and without dog, ‘Engagement-and-meaning of life’ as well as ‘Physical activity with the dog’ were the most discussed roles. The ‘Sleep or wake up’ role was the least discussed role across three cases. In the other categories, they were seven advantages and 10 inconvenients that were mentioned for canine assistance. For home care support, the presence of NSD has more positive impacts on both the person with dementia and their caregiver compared to the presence of a companion dog; the presence of a NSD results in the person with dementia accessing more indoor and outdoor public sites than with a companion dog; and dyads with a dog are informally socially engaged more frequently than those with no dog.

## Introduction

Family caregivers are often relied upon to help individuals with dementia to manage sleep deficiency (McCurry et al., 2006), and to remain active, connected to their communities (Eales et al., 2015), and safe amidst declining cognition and functional abilities. Family caregivers can play several roles in numerous areas: assistance with household tasks, self-care tasks, and mobility; provision of emotional and social support; health and medical care; advocacy and care coordination; and surrogacy. Each area has multiple tasks and activities (Schulz & Eden, 2016). Independent from these aeras, caregivers do continual problem solving, decision making, communicating with others (family members and health and human service professionals), and constant vigilance over the care recipient’s well-being (Gitlin & Wolff, 2012). In a study with 606 caregivers of persons with dementia, cancer, diabetes, or frail elderly, dementia caregivers were most likely to help by dealing with the care recipient’s incontinence or diapers compared to cancer caregivers to provide more help getting in and out of a bed, chair, or toilet and by feeding the care recipient (Kim & Schulz, 2008). Both cancer caregivers and dementia caregivers are equivalent to frail elderly caregivers to help in getting dressed, bathing or showering, and giving pills, medications, or injections. Cancer or dementia caregivers were similar to arrange or supervise outside services for care recipients than diabetes or frail elderly caregivers (Kim & Schulz, 2008).

The present research was initiated by a caregiver and stakeholder working with the community organization Early Onset Dementia Alberta Foundation (EODAF, 2020). A voice for families affected by early onset Alzheimer’s and dementia (<65 years old), EODAF had observed home support being provided by companion dogs paired with persons living with dementia. EODA contacted a Canadian researcher known for publications on service dogs (Vincent et al., 2015, 2017a, 2017b, 2019) to study the effectiveness of canine assistance as an innovative nonpharmacological support option for caregivers and community-dwelling persons with mild to moderate dementia. Since there was still no scientific evidence supporting the use of neuro service dogs for people with dementia in 2018, the Canadian Alzheimer’s Foundation funded a research project in 2018 to examine canine assistance for caregivers and their loved ones with dementia in a home support setting. The **goal** of the present study was to examine how canine assistance may support family caregivers and persons with dementia and to document and compare two modalities of home care support (neuro service dog and companion dog) from the perspectives of family caregivers.

### Scientific evidence after 2018

A systematic review of trained assistance dogs for people with dementia was published by Marks and McVilly (2020). From the 24 papers included in their review, there were no papers that specifically addressed the issue of assistance dogs for those with young onset dementia living at home. Interventions with dogs were done in nursing homes, day care facilities and long-term facilities or hospital geriatric psychiatric unit. Dog obedience, therapy dog and pet dog were used in 21 studies as trained assistance or service dog was used in three studies. None of the paper addressed the allocation of an assistance dog to a particular participant. The length of exposures time was inconsistent, short (30–60 minutes visit) or intermittent basis, from a year part-time basis to 12 weeks. There is scientific evidence that dog-assistance intervention with the people with dementia in these institutions has improved their mood (11 studies), pro-social behaviors (10 studies), daily activities and quality of life (13 studies), cognitive awareness in six studies (enhanced skills in learning new things, completing new tasks, mental and physical stimulation, enhanced memory). Also, three studies with phenomenological hermeneutic approaches found emerging themes such as love and attachment, meaning of life, self-worth and other existential issues (Marks & McVilly, 2020). In 2021, Ritchie et al., (2021) have published findings of the pilot phase of the Dementia Dog Project (2013–2015) realised in Scotland. The pilot project focused on training and placing four dementia assistance dogs with couples where one partner had a diagnosis of dementia. Using a realistic evaluation, Initial programme theory and Theory of Change (Weiss, 1995), they focussed on the context, mechanisms, and outcomes (CMO) and pointed out why an approach may work in some situations but not others (one case had withdrawn during the pilot). Unlike the beginning of their study, the final CMO configurations for the Dementia Dog Project is now illustrated with variables in text format and there are arrows for potential associations between variables (Ritchie et al., 2021). There are 2 variables for Context (Dog is trained to meet the needs of the couple; Changing context – progression of dementia and responding to changing need). Those are linked to three variables namely the Mechanisms (Human–animal bond, Relationship dynamics, and Motivation–the responsibility of caring). Those mechanism may produce six possible Outcomes. One mechanism may be associated to three or four outcomes. The “**human-animal bond**” is the key concept within animal assistive intervention that include the relationship between the human and animal. It is associated to four outcomes such as “emotional support for the couple, improved communication between the couple and social settings, reduction in caregiver stress and worry and care and support for the person with dementia, their partner and the dog”. The bond is based on trust and reciprocity, which positively influence the health and well-being of both human and animal. In case #4, there were no bond with the person with dementia only with the caregiver; this justifies the withdrawal from the pilot project after five months. The mechanism “**relationship dynamics**” related to the role the dog takes within the couple and the flexibility of that role as the context change. It is associated to four outcomes including “**Maintenance of routine**”. For three couples, the dog fitted easily in their life and they spoke about the dog as fitting in their relationship (e.g., in focussing on the dog it helps to facilitate sensitive conversations about the future). In case #2, the person with dementia had to move to the care home but the dog stays with the spouse. The dog continued to act as a buffer between the couple and it helps the spouse to have more positive visits to the care home. The mechanism “Motivation - responsibility of caring” is the motivation the dog stimulates in the couple because the dog is reliant on the couple for care (to go out and walk the dog daily). It is associated to three outcomes including “increase in activity”. There is also improvement in mood because it increases the social activities of the couple. Dog by nature are driven by routines and this will naturally instigate a routine in the couples.

Marks and McVilly (2023) are the first that published results about assistance dogs for people with early onset dementia only in Australia. Their study involved 14 people of 53–65 years old matched with trained assistance dogs over a two-year period. Interviews with family carers were conducted. Training tasks included: head on lap (on demand), fetch the carer, dog follows participant throughout the house unless commanded otherwise, find object (mobile telephone, glasses, etc.), stop at kerb, and tug and response games (i.e., where the dog waits for a command to take a toy and then waits for a commend to release the toy). All dogs were trained to walk in harness, and in the usual skills expected of Seeing-eye (guide) dogs for the blind. They presented their results according to the three key mechanisms presented above by Ritchie et al. (2021), that are human–animal bond, relationship dynamics, and responsibility for caring and carer wellbeing. The 11 participants also experienced the same six outcomes, except for three persons with dementia that went into care during the study.

### A dementia dog is a neuro service dog

The Dementia Dog (2022) organization supported by Alzheimer Scotland indicates that, “dementia assistance dogs are highly trained dogs, helping their forever family with specific tasks such as fetching medication pouches. The dogs have full public access rights and live with the family at home, full time.” Neuro service dog are intended persons with dementia, Alzheimer’s, Parkinson’s, and brain injury (Wilderwood Service Dogs, 2020). Paws for life USA-Services and therapy dogs (2022) announces Neurological Service Dogs (Dementia, Alzheimer’s, Parkinson’s, Huntington’s, Brain Injury, Lupus, Narcolepsy and Psychological Disabilities). Krause-Parello et al. (2016) and Taylor et al. (2015) claimed that service dogs are exhaustively trained to respond precisely to specific disabilities of their owners and are typically allowed entry into public facilities under the protection of the Americans with Disabilities Act (ADA). Emotional support dogs and companion dogs (pet dog) do not undergo specific training to provide aid to individuals or for public access. Only services dogs are trained for public access and specific tasks to support the needs of the person. Service dogs are commonly known to assist individuals with physical disabilities, such as paralysis, blindness, diabetes, and seizures. Psychiatric service dogs attend to individuals with diagnoses such as schizophrenia, bipolar disorder, obsessive–compulsive disorder, panic and anxiety disorders, as well as PTSD.

### Rationale for the study

In Canada, the estimated annual cost of dementia is US$10.4 billion (Chambers et al., 2016, p. 40) and of unpaid caregiving is US$25 billion (Hollander et al., 2009). A 2012 survey (Sinha, 2012) found that, of the 8.1 million Canadian carers (28% of the national population), 44% were between the ages of 45 and 64 years, 10% provided more than 30 hours of support a week, 60% continued to work while providing care, and 25% were simultaneously caregiving and child-rearing. Furthermore, approximately 50% of family caregivers cared for seniors with health conditions (Turner & Findlay, 2012), of which nearly half a million people had dementia (often a parent or in-law) (Eales et al., 2015). The overwhelming majority of caregivers (89%) offered support for a minimum of one year, with 50% doing so for at least 4 years (Sinha, 2012).

Supporting family caregivers is a national public health priority given their essential role in the healthcare system (Hollander et al., 2009) as acknowledged in the Health Council of Canada (2012) “Seniors in need, caregivers in distress” report. Caregivers, however, require support to foster resilience (adapt well in the face of adversity) and ensure that they can continue to provide care while maintaining their own wellbeing. Despite most caregivers reportedly being in good, very good, or excellent physical and mental health, caring can take its toll and be burdensome (Brémault-Phillips et al., 2016; Butler-Jones, 2010; Eales et al., 2015; Health Council of Canada, 2012; Sinha, 2012). Caregivers are at increased risk of physical, emotional, and financial burden if: (1) they provide more than 21 h per week of care; (2) care for persons with depression, cognitive decline, behavioural change; or (3) care for persons with terminal conditions (Butler-Jones, 2010; Canadian Institute for Health Information, 2010; Health Council of Canada, 2012; Hollander et al., 2009; Sinha, 2012). Stress can result in health challenges, social isolation, loss of income, and family conflict (Dumont et al., 2009; Health Council of Canada, 2012; Stajduhar et al., 2010). The strain on family caregivers is anticipated to intensify given the aging population (Dudgeon, 2010; Eales et al., 2015; Smetanin et al., 2009).

### Specific objectives

Given the limited evidence of canine assistance for home care support in 2018, our study had two objectives:

1. To examine the impact of canine assistance on engagement, meaning of life, socialisation, sense of direction, physical activity, sleep and other participation activities for both the person with dementia and his/her caregiver.
2. To examine the informal acceptance of canine assistance in public places

## Methods

### Research approach

An exploratory comparative case study research design (Bartlett & Vavrus, 2017) was conducted. This research approach is relevant if it is important of comparing the conceptualization in different cases, if using theoretical constructs, and if focusing on tracing the phenomenon of interest in a study (Bartlett & Vavrus, 2017). The cases in our study correspond to three different groupings. Each grouping consisted of dyads of a caregiver, a person with mild to moderate dementia, and either (1) a neuro service dog, (2) a companion dog or (3) no dog. Chigbu (2019) suggests using hypotheses and visual representations to capture differences in qualitative results, in using diagrams with themes for example. We have done that for the three cases. Our general hypothesis is: there are different conceptualisations about having a neuro service dog, a companion dog or no dog between the three groups. Given the purpose of our study to compare two modalities for home care support, we have formulated hypotheses to show differences between the three cases. H1: The presence of a neuro service dog (NSD) has more positive impacts on both the person with dementia and their caregiver compared to the presence of a companion dog. H2: The presence of a NSD results in the person with dementia accessing more indoor and outdoor public sites than with a companion dog. H3: Dyads of persons with dementia and caregivers with a NSD or a companion dog will be informally socially engaged more frequently than those with no dog. This project received three ethical approvals, one from Université Laval, one from University of Alberta and one from Centre intégré universitaire de la santé et des services sociaux de la Capitale nationale (UL 2018–185, UofA Pro00083915, CIUSSS-CN 2021-2230).

### Recruitment of study participants

Case 1 aimed to include six to 10 NSD and dyads caregiver/person with dementia. Recruitment was done by the research coordinator through the Maryville Tennessee-based Wilderwood Service dogs organization. Case 2 aimed to include 20 to 30 dyads caregiver/person with dementia with a companion dog. This group was recruited online through the Canadian Alzheimer associations’ eight provincial divisions and was overseen by *Early Onset Dementia Alberta*. Also, more than 150 associations caring for people with dementia or for caregivers in the US and Canada were contacted twice by email and asked to distribute the study invitation. Case 3 aimed to include 20 to 30 dyads of caregiver and person with dementia who had no dog, but were on a waitlist for a NSD at Wilderwood Service dogs. In addition, the records of a hospital in the Quebec City area were searched to identify patients with cognitive disorders associated with dementia and invitations to participate in the study were sent.

Selection criteria were: 1-Caregivers must have a companion dog for more than one year or must have had a NSD for a minimum of three months or be on a waiting list for a NSD or the NSD must have died in the last year. 2-The caregiver must be willing to discuss their experience with a dog, or reason for wanting to have one or not. 3-The person with dementia must be community-dwelling and residing with the caregiver, and 4-The person with dementia must be in the mild or moderate stage of the disease.

### Data collection procedure

Data were collected through a telephone call of 45–60 minutes. First the interview was conducted and then the sociodemographic and caregiver burden questionnaires were done. A pre-tested interview guide was followed. Interviews were conducted by co-authors FD (*n* = 32), AR (*n* = 20) and MR (*n* = 4). Data collection began in 2018 and concluded in December 2021. Recordings were manually transcribed by students in psychology (*n* = 21) and occupational therapy (*n* = 9), and by co-authors AR (*n* = 20), MR (*n* = 4) and FD (*n* = 2).

### Qualitative data

We worked with a **phone interview guide** based on one used for veterans with PTSD who had a service dog (Lessard et al., 2018). The phone call was undertaken by the caregiver, and with the person with dementia if possible. The person with dementia joined the caregiver at the beginning of the interview when talking about the dog’s roles and tasks. There were four questions for the caregiver and two for the person with dementia regarding: (1) The use of the dog in rural or urban contexts (reasons for use, tasks, and activities), frequency of use, and intensity of use. If no dog: the participant was asked: “Why do you want a dog or why not?”; (2) Advantages with the use of the dog; (3) Disadvantages, inconveniences, or obstacles with the use of the dog. If no dog, “what are the expected advantages and disadvantages of having a dog?”; and (4) Recommendations for the maintenance of the NSD program in the future. (1) What do you do with your dog? (2) And what is your dog doing for you?

### Sociodemographic variables and co-variables

For sociodemographic variables, there were four questions about the caregiver, seven about the person with dementia and five related to the dog. The **Caregiver’s Burden Scale (CBS)** was completed at the end of the phone interview. This questionnaire measures the perception that caregivers have of their partner’s capabilities and the burden on caregivers (Dumont et al., 2009). The perception of capabilities was comprised of five questions about what daily activities or mobility the person with dementia can accomplish. The 16-item scale (of burden) asks caregivers to circle the score that best reflects their situation on a 4-points scale from ‘Not at all’ to ‘Very often’. A higher score suggests a stronger burden on the caregiver. In the Dumont et al. (2009) study of 167 participants, the questionnaire was shown to have high internal consistency (Cronbach’s alpha = .95), good construct validity as items were associated to the two predicted factors (61% of the variance explained, with a KMO of .93), and convergent validity showed good correlations to other questionnaires.

### Data analysis

Descriptive statistics were conducted for sociodemographic data, CBS responses, and the coded interview responses. There are total, frequency, mean, median, standard deviation, total score, mean item per item score, number of quotes, percentage of quotes per category.

A qualitative approach was used to analyze interview data (Baker et al., 1992). Using an inductive approach (Fontana & Frey, 2000), 10 transcripts were read in their entirety. A second reading identified meaning units (excerpts from the text that correspond to an idea) that were categorised according to the four topic questions (categories). Meaning units have inspired the title for themes and subthemes which were used to code text from interviews using NVivo 12 (released in March 2020). Explanations for themes were generated and sub-themes were labelled using terms from the grey literature of NSD. A first draft of the codes was done during the summer of 2020, by the principal investigator, MR and one student in occupational therapy. A first interview was individually coded and then compared. Sub-themes were specified. When the same exercise was repeated with another interview, the inter-rater agreement was higher than the first pre-test. Two sub-themes were also redefined. The final list of codes was numbered. For example, the first category is 1*. Roles and usages of the dog.* 1.1 *Engagement-and-meaning of life* is one of the six themes of this first category. And those are the sub-themes for that theme: 1.1.1 *Obligations, responsibilities, or valued occupation towards the dog*, and 1.1.2 *Companionship and solicitude*. Text was intended to be encoded at the last level.

The 10 interviews were initially entered into NVivo by MR. The research coordinator coded the remaining 46 interviews. Extraction of data was done quantitatively (number of persons, number of quotes, percentage of quotes per category) and qualitatively (relevant quotes). In order to consult the data without the software, qualitative data was extracted from the NVivo data base into three distinct Word documents (Case 1, Case 2, Case 3). A table of content on the first page allowed the efficient retrieval of the sub-themes containing quotes from caregivers and persons with dementia associated with the date of the interview. The principal investigator read all the three cases, copied and pasted pertinent quotes supporting the sub-themes presented in this manuscript. Quantitative data was extracted from the data base of NVivo into EXCEL. This allowed the calculation of statistics for themes and sub-themes for compare their “weight of quotes” across the three cases.

## Results

### Sociodemographic

We conducted 56 interviews with caregivers and persons with dementia who lived together. Table 1 presents sociodemographic for caregivers and statistics for the three cases. There were five caregivers (mean age 54.8 years) who had an NSD, 28 caregivers (mean age 63.6 years) who had a companion dog, and 23 caregivers (mean age 63.8 years) who did not have a dog. Among the 56 caregivers, 39 were women, 46 were spouses, and 17 were still working. With respect to the capabilities of participants with dementia, there was a tendency for those from the no dog group to be confined to their chair or bed: 73% *spent more than half of the day in bed or in a chair* (*p* = .90) and 27% were *practically completely confined to bed or chair* (*p* = .88). Results on the Caregiver’s burden scale indicated that there was no difference between the three groups for 14 of the 16 items. The sum of the items indicates greater severity of the burden as one approaches the maximum of 64. There was a tendency (*p* = .061) for caregivers in the NSD group to be experiencing less burden than the other two groups (NSD: 23.2/64, companion dog: 32.8/64 (post hoc *p* = .026) and no dog: 34.4/64 (post hoc *p* = .023)). According to Table 1, the proportion of men and women is different between groups (*p* = .42), as well as CBS item 4 (*p* = .31) and item 8 (*p* = .18).

**Table 1.** Sociodemographic information of caregivers who have done the interviews (*N* = 56).

	Neuro service dog		Companion dog		No dog		Kruskal Wallis test^a^
	*n* = 5		*n* = 28		*n* = 23		
Caregiver	*n*	%	*n*	%	*n*	%	*p-value*
Men	3	60	5	16	9	39	.042
Women	2	40	23	84	14	61	.042
Relation with the person with dementia							
Spouse	4	80	22	79	20	87	.739
Dating	1	20	0	0	1	4	
Children	0	0	5	18	2	9	
Brother or sister	0	0	1	3	0	0	
What is your main occupation?							
Work	3	60	8	29	9	39	.090
Caregiver	0	0	10	36	7	30	
Retired	2	40	5	18	7	30	
Volunteerism	0	0	1	4	0	0	
Medical leave	0	0	2	7	0	0	
Mean age in years (SD) perception of capabilities mean of % (SD in %)	54.8 (11.6)		63.6 (8.5)		63.8 (9.9)		.236
- Can run errands without assistance mean	60 (55)		46 (51)		55 (51)		.780
- Can manage daily activities without help	100 (0)		71 (46)		73 (46)		.396
- Requires assistance to move around inside at home	0 (0)		18 (39)		27 (46)		.364
- Passes more than half of the day in bed for in a chair	20 (45)		57 (50)		73 (46)		.090
- Is practically completely confined to bed or chair	0 (0)		7 (26)		27 (46)		.088
Caregiver’s burden scale mean (SD) 16 items, score/64	23.2^b,^ ^c^ (4.7)		32.8 (10.2)		34.4 (11.7)		.061
- Item 4: Do you ever feel unable to go on? 1- Never to 4-very often	1.0 (.0)^d,^ ^e^		1.9 (.9)		2.1 (1)		.031
- Item 8: Do you ever feel that you are no longer capable of caring for the ill person? 1- Never to 4: Very often, in mean (SD)	1.0 (.0)^f^		1.4 (.7)^g^		1.9 (.9)		.018

^a^Statistical difference between groups if *p* ≤ .05.

Post hoc (Mann Whitney):

^b^NSD different from Companion dog (*p* = .026).

^c^NSD different from No dog (*p* = .023).

^d^NSD different from Companion dog (*p* = .045).

^e^NSD different from No dog (*p* = .010).

^f^NSD different from No dog (*p* = .040).

^g^Companion dog is different from No dog (*p* = .033).

Table 2 presents sociodemographic for persons living with dementia and statistics for the three cases. Among the 56 people with dementia, 31 were women, 27 had a mild dementia and the most frequent causes of dementia was Alzheimer (*n* = 15) and Parkinson (*n* = 14). Average age differed (*p* = .19) between the groups: 53.6 years (NSD), 72.6 years (companion dogs) or 68.7 years (no dog). However, there was no difference in the number of years since their dementia diagnosis. Four of the five people who have a NSD lived in the USA (where the training school for NSD is located) while the fifth lived in New Brunswick, Canada (went to a Canadian training school for service dogs). The dyads who either had a companion or no dog lived mostly in Quebec (33), in five other Canadian provinces (10), and in the United States (7). Participants lived in cities (20), suburbs (20), countryside (10) or on a farm (3).

**Table 2.** Sociodemographic information of persons with dementia and of their dogs (*n* = 56).

	Neuro service dog		Companion dog		No dog		Kruskal Wallis test^a^
	*n* = 5		*n* = 28		*n* = 23		
Person with dementia	*n*	%	*n*	%	*n*	%	*p*-value
Men	2	40	10	38	13	56	.575
Women	3	60	18	62	10	44	.575
Level of dementia							
Mild	4	80	13	48	10	44	.329
Moderate	1	20	15	52	13	56	
Causes of dementia							
Alzheimer	2	40	7	25	6	26	.080
Parkinson	1	20	2	7	11	48	
Vascular	0	0	2	7	1	4	
Aging	0	0	2	7	1	4	
Other	1	20	7	28	3	13	
Unknown	1	20	8	29	1	4	
Where do you live?							
Quebec	0	0	15	54	18	78	.010^a^
USA	4	80	5	18	2	9	
Saskatchewan	0	0	3	11	1	4	
Ontario	0	0	2	7	1	4	
NB	1	20	0	0	0	0	
BC	0	0	1	4	1	4	
Manitoba	0	0	1	4	0	0	
Do you live in?							
City	2	40	10	36	8	35	.777
Suburban	1	20	10	36	9	39	
Rural	2	40	5	18	3	13	
Farm	0	0	0	0	3	13	
What is the dog size?							
Small	0	0	8	29	—	—	.504
Medium	4	80	11	39	—	—	
Large	1	20	7	25	—	—	
Other animal in your house?							
Yes	5	100	8	32	6	26	.006^a^
No	0	0	17	68	17	74	
If yes, what type?							
Another dog(s)	2	40	2	20	1	17	.799
Cat(s)	2	40	6	60	5	83	
Other	1	20	2	20	0	0	
Mean age in years (SD)	53.6 (12.0)		72.6 (11.3)		68.7 (10.4)		.019^a^
Number of years since diagnosed with dementia (SD)	3.2 (1.3)		3.7 (2.4)		5.6 (5.9)		.838
Mean age of the dog (SD)	2.4 (.9)		5.4 (3.7)		—		.084
Number of years since the acquisition of the dog (SD)	1.2 (1.1)		4.9 (3.8)		—		.013^a^

^a^Statistical difference between groups if *p* ≤ .05*.

Table 2 reveals that half of the dogs were of medium size. The mean age of the NSD was 2.4 years old and 5.4 for the companion dogs which reveals a tendency for the NSD to be younger than their counterparts (*n* = .084). The acquisition of the NSD was more recent (1.2 years before the interview) compared to the companion dogs (4.9 years) (*p* = .013). Table 2 presents results and statistics for the three groups. People with an NSD all have other pets at home which is more than the other two groups (*p* = .006). Also, there was a difference in the participants’ origin mainly because none of those with an NSD come from Quebec compared to the other groups (*p* = .010).

### Overview of the coding

**Figure 1.**
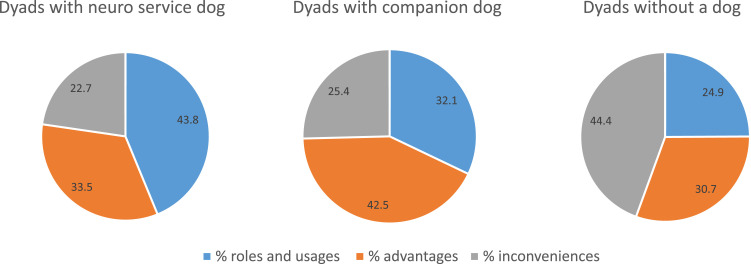
Portrait of the distribution of the codification according to the dyads. The Roles and usages (blue – 43.8%) take a greater place in the discussion of those dyads having a neuro service dog, whereas those having a companion dog talked more about the Advantages (grey – 42.5%) of having a dog and, those not having a dogs talked more about the Obstacles and disadvantages (orange – 44.4%). Legend: On the left, the dyads that live with a neuro service dog (*n* = 5), in the center the group that lives with a pet dog (*n* = 28) and on the right the group that does not have a dog (*n* = 23). In blue (the roles and uses of the dog); in grey (the advantages of having a dog); in orange (the obstacles and disadvantages) and, in yellow (future recommendations for home support).

Using NVivo color-mapping, we were pleased to find that there was a great difference in codification according to the group, in what dyads said about their NSD, companion dog and having no dog. The category “roles and usages” occupy more space in NSD case while the category “advantages” takes more space in companion dog case. We see that for the group without a dog, it is the “obstacles” category which takes more place (Figure 1). Tables 3–5 provides all themes and subthemes for each category with statistics for participants, quotes, and percentage of quotes, in the next three sections.

**Table 3.** Five roles and usages of having a dog, documented by caregivers living with a person with dementia, with a neuro service dog, companion dog or no dog (*n* = 56).

Themes	Dyads with neuro service dog			Dyads with companion dog			Dyads without a dog		
Subthemes	*n*	q	% q./c	*n*	q	% q./c	*n*	q	% q./c
Category of roles and usages	5	81	100	28	197	100	20	65	100
**Engagement-and-meaning of life**	**5**	**17**	**21**	**27**	**74**	**38**	**15**	**28**	**43**
Obligations, responsibilities, or valued occupation towards the dog	5	10	12	24	50	25	10	11	17
Companionship and solicitude	4	7	9	14	24	12	10	17	26
**Socialization** ^H2 andH3^	**5**	**21**	**26**	**17**	**25**	**13**	**1**	**1**	**2**
See people	5	19	23	14	19	10	1	1	2
Develop significant relationship with others	2	2	2	5	6	3	0	0	0
**Physical activity with the dog** ^H1^	**5**	**13**	**16**	**26**	**62**	**31**	**16**	**24**	**37**
Play with the dog	2	4	5	9	11	6	4	4	6
Walk with the dog	5	9	11	24	51	26	15	20	31
**Help with sense of direction** ^H1^	**4**	**21**	**26**	**14**	**22**	**11**	**3**	**3**	**5**
Great confidence in the dog	3	6	7	9	12	6	0	0	0
Keep the person safe and take the person to destination	4	15	19	8	10	5	3	3	5
**Sleep or wake up**	**1**	**2**	**2**	**9**	**12**	**6**	**1**	**1**	**2**
Time it takes to fall asleep, or time spent sleeping at night	1	1	1	5	5	3	0	0	0
Number and duration of awakenings during the night	0	0	0	1	1	1	1	1	**2**
Alarm clock	1	1	1	5	6	3	0	0	0
**Other unexpected usages** ^H1^	**3**	**7**	**9**	**2**	**2**	**1**	**5**	**8**	**12**

q. = quotes.

% q./c. = percentage of quotes of this category.

H1 = the first exploratory research hypothesis is confirmed: the presence of a NSD has more positive impact - on this theme-than the presence of a companion dog.

H2 = the second exploratory research hypothesis is confirmed for this theme: The presence of a NSD results in the person with dementia accessing more indoor and outdoor public sites than with a companion dog.

H3 = […] for this theme: Dyads of persons with dementia and caregivers with a NSD or a companion dog are informally socially engaged more frequently than those with no dog.

**Table 4.** Seven advantages of having a dog, documented by caregivers living with a person with dementia, with a neuro service dog, companion dog or no dog (*n* = 56).

Themes	Dyads with neuro service dog			Dyads with companion dog			Dyads without a dog		
Subthemes	*n*	q	% q./c	*n*	q	% q./c	*n*	q	% q./c
Category of advantages	5	62	100	28	261	100	22	80	100
**General feeling of security**	**5**	**18**	**29**	**26**	**89**	**34**	**14**	**29**	**36**
Decreased anxiety or reduced stress	4	8	13	21	42	16	9	13	16
Developing friendship with the dog	5	10	16	21	47	18	11	16	20
**Well-being and quality of life**	**4**	**19**	**31**	**23**	**74**	**28**	**9**	**24**	**30**
Have life significant	3	6	10	18	30	11	5	6	8
Happiness and other positive feelings	4	13	21	21	44	17	9	18	23
**Monitoring loved one’s sleep and napping**	**3**	**13**	**21**	**17**	**33**	**13**	**4**	**6**	**8**
Dog goes to bed with the loved one	3	5	8	14	23	9	2	2	3
Dog allows better sleep to the loved one	3	6	10	6	6	2	2	2	3
Dog allows better sleep to the caregiver	1	2	3	3	4	2	2	2	3
**Independence** ^H1^	**5**	**6**	**10**	**20**	**40**	**15**	**6**	**11**	**14**
Opportunity to relax or some headspace to think to something else	3	3	5	7	9	3	4	5	6
Less anxiety for the caregiver	1	1	2	12	19	7	3	3	4
Allows the caregiver to leave home for few hours	2	2	3	12	12	5	3	3	4
**Positive economic impacts** ^H1^	**1**	**2**	**3**	**5**	**5**	**2**	**0**	**0**	**0**
Allows the caregiver to keep a job	1	2	3	5	5	2	0	0	0
Allows the caregiver to have a career	0	0	0	0	0	0	0	0	0
**Positive focus on the dog**	**1**	**1**	**2**	**11**	**13**	**5**	**4**	**5**	**6**
**Reminders coming from the do**g^H1^	**1**	**1**	**2**	**1**	**1**	**0**	**3**	**3**	**4**

q. = number of quotes.

% q./c. = percentage of quotes of this category.

H1 = the first exploratory research hypothesis is confirmed: the presence of a NSD has more positive impact - on this theme-than the presence of a companion dog.

**Table 5.** Ten inconveniences, disadvantages, and obstacles of having a dog, documented by caregivers living with a person with dementia, with a neuro service dog, a companion dog, or no dog (*n* = 56).

Themes	Dyads with neuro service dog			Dyads with companion dog			Dyads without a dog		
Subthemes	*n*	q	% q./c	*n*	q	% q./c	*n*	q	% q./c
Category of inconveniences	5	42	100	27	156	100	22	116	100
**Reaction of employees or clients**	**5**	**8**	**19**	**12**	**17**	**11**	**6**	**8**	**7**
Fear of the dog	0	0	0	1	1	1	2	2	2
Allergies to dog	0	0	0	2	2	1	2	2	2
Ban on dogs in shops^H2^	3	3	7	7	12	8	3	3	3
Bad people’s reaction to dogs	3	5	12	2	2	1	1	1	1
**Maintenance**	**4**	**12**	**29**	**17**	**42**	**27**	**17**	**61**	**53**
Hair in the house because of the dog	1	2	5	2	3	2	4	4	3
Cleaning the house	0	0	0	3	3	2	6	7	6
Maintenance or daily care of the dog	1	3	7	13	17	11	14	39	34
Annual visit to the veterinarian	2	2	5	6	7	4	5	6	5
Food, tricks	2	3	7	8	9	6	4	4	3
Dog grooming	2	2	5	2	3	2	1	1	1
**Dog’s health problem or bad behavior**	**2**	**8**	**19**	**17**	**26**	**17**	**7**	**10**	**9**
Too active for the person or hyperactive	2	6	14	14	18	12	4	5	4
Fearful dog (thunderstorm, door slamming)	2	2	5	6	7	4	1	1	1
Destructive dog	0	0	0	1	1	1	4	4	3
**People’s reaction in the street**	**1**	**1**	**2**	**2**	**2**	**1**	**0**	**0**	**0**
**Dog’s needs for break time (meal, sleeping, toileting sites)**	**2**	**2**	**5**	**6**	**6**	**4**	**0**	**0**	**0**
**Acquisition and training (time, cost)**	**2**	**2**	**5**	**3**	**2**	**1**	**6**	**7**	**6**
Obedience training for pet dogs	0	0	0	1	1	1	2	2	2
Training in a service dog school or not	2	2	5	1	1	1	3	5	4
**Extra care and cost (surgery, medication, medical monitoring)**	**2**	**3**	**7**	**9**	**12**	**8**	**6**	**8**	**7**
**Dealing with the absence of the dog (death of the dog, placement in residential care)**	**3**	**3**	**7**	**17**	**28**	**18**	**4**	**5**	**4**
**Inappropriate focus on the dog**	**0**	**0**	**0**	**6**	**10**	**6**	**1**	**1**	**1**
**Fears and disinterests of having a dog**	**3**	**3**	**7**	**7**	**11**	**7**	**11**	**16**	**14**

q. = quotes.

% q./c. = percentage of quotes of this category.

H2 = the second exploratory research hypothesis is confirmed for this theme: The presence of a NSD results in the person with dementia accessing more indoor and outdoor public sites than with a companion dog.

### Roles and usages of the dog

Table 3 shows the six roles and usages of the dog with the percentage of quotes for each case. Also, it points out the four specific themes where the research hypothesis H1, H2 and H3 are supported in favor of the NSD group. When comparing the cases with percentage of quotes in the category of roles and usages, Socialisation and Help with a sense of direction were the most addressed roles for dyads with the NSD. For dyads with companion dog and without dog, Engagement-and-meaning of life as well as Physical activity with the dog were the most discussed roles. People without dogs mentioned that they would like to walk with a dog as it would give them more exercise. The Sleep or wake up role was the least discussed role across three cases. Other unexpected usages were reported by dyads with NSD and without dog, but are miscellaneous: It is either a matter of the NSD making sure that the person with dementia turns off the stove after cooking, the NSD helping the person with dementia balance on stairs and during walks outside, or approaching the person with dementia in case of loss of balance; and finally, the NSD bringing or pointing out warm clothes when it is cold.

#### Socialization role

Neuro service dogs are trained to contribute to socialisation. Staying socially connected is the principal recommendation from the Alzheimer Society of Canada (2022). From the first onset of symptoms of dementia, socialisation is encouraged so as “to make the most of your daily opportunities to socialize, to practice a random act of kindness, find time to volunteer, to combine social interaction with an activity and to maintain old friendships and make new ones”. Walking alone with a dog may encourage people to approach them and talk to them about their dog, thus keeping them connected to society. This is it, H3 is confirmed. However, if the dog is not a NSD (i.e., not wearing a vest that certifies it is a dog trained to support the person and allowed to public facilities), it may be difficult to enter businesses, especially churches, clinics and restaurants with a dog. We had expected socialization to be more intense with an NSD because it is allowed to accompany its owner inside public or commercial buildings. This is it, H2 is confirmed. We also observed that the sub-theme “See people” is more discussed in dyads with a NSD than the other dyads (NSD 23%; companion dog 10%; no dog 2%) (Table 3). Furthermore, “Developing significant relationships with others” seems possible when having a dog with you (NSD 2%, companion dog 3%, no dog 0%). This is illustrated in the following quotes.

“Oh! My life is definitely better with the dog. I’m not usually a social butterfly by any means. I hope to talk to a lot of people, but when I’m out with the dog I have a lot of people to talk to me. I get to meet the neighbors … I got a lot of friends in this neighborhood because of the dogs. You get to meet a lot more people in general.” (NSD, 30-09-2020, person with dementia)

“So the ability to connect words in a conversation is difficult for him. When he talks about the dog, it gives him something familiar to talk about and something that he knows. [He is] not put on the spot so much and so as far as socialization goes, I would say it helped because [..]. he has a topic to talk about, that he feels confident with.” (NSD, 19-02-2019, caregiver)

“The people at church are very impressed with her [the dog], because I sing in the choir, and she has to be right there with me, so she's in the choir when we’re performing. And one day, a lady that was [...] standing next to me, who has Alzheimer’s, was losing her balance, and Teagan had the wherewithal to turn around and nuzzle her and get her back onto the stool. […] She’s very alert to everything around her, it’s amazing […]. This particular church is fairly new to both of us and these folks have just adopted her, hum to the individual. Every person that is in that congregation has adopted her.” (NSD, 25-01-2019, person with dementia)

“I think so. My husband was always a very private quiet kind of man. I think the dog has brought out more of conversations with him. He is able to talk to the dog and use the dog almost as a tool for conversation... our neighbors that are here full time engages with the dog which helps my husband engage with them.” (Companion dog, 18-05-2019, caregiver)

#### (Role of) help with sense of direction

Neuro service dogs are taught to assist with a sense of direction, which is defined by “an ability to know roughly where you are, or which way to go, even when you are in an unfamiliar place” (Educalingo, 2022). Because the NSD knows where it lives, has learned certain routines for walks, and sometimes certain specific routes in public places, it can compensate for the loss of a sense of orientation in people with early dementia. This benefit is reported more frequently by dyads with NSD (NSD 26%; companion dog 11%; no dog 5%). In Table 3, we can see that the sub-theme “Keep the person safe and take the person to destination” is more supported for dyads with a NSD than the subtheme “Great confidence in the dog”. Those quotes support this idea.

“They were training her, when we say home and she would know where our house was. So this one we are working on it. There is always a work in progress.” (NSD, 19-02-2019, caregiver).

“Like if she got confused or lost in a store, he will hunt for one of us to take us to the other person.” (NSD, 10-04-2019, caregiver)

“Well, it’s sure that if it’s adapted for him, he could even go walking alone with the dog. Because if he freezes, the dog would stop too. ... So, if he had a dog, he could make a little gesture on the harness and the dog would accompany him. It would be an easy command, ... just by making a gesture, well the dog would go ahead and if he wants to stop it’s the same thing, he makes a gesture and the dog would stop. So that would be a big advantage.” (No dog, 21-06-2021, caregiver)

#### Engagement-and-meaning of life (role in)

Healthy dogs do not need to be trained to foster Engagement-and-meaning of life, this is one of dog instinctive quality. The fact that the dog needs to be cared for allows the person with dementia to continue to feel engaged in the role of dog owner. For some, it is one of the last roles that give meaning to their life at home despite losing their cognitive faculties over the course of dementia. This role is strongly supported in the cases especially in dyads with companion dogs and no dog (NSD 21%, companion dog 38%, no dog 43%). In Table 4, we can see that caregivers with companion dogs express more concerns with the sub-theme “Obligations, responsibilities or valued occupation towards the dog”. Here are supporting quotes from two groupings:

“I’d literally be lost somewhere without mine [NSD]. God, I don’t know where I would be. And today, Sky basically dragged me to kickboxing and dragged me home. It’s because she knows the routine and that’s what we do. So, Tuesday was a wired day. She knows when I’m off. And she just got to keep the routine going? And, that’s so important ... I mean, routine is everything […] because their dog is going to keep in there, that’s a definitely great thing.” (NSD, 30-10-2020, person with dementia)

“I would recommend one, yes. Just for the purpose. It gives you purpose; gets you up, keeps you motivated or active. And he’s a good companion, gives you emotional support and yeah I would recommend it to anybody if they had a chance to get one.” (NSD, 06-09-2019, person with dementia)

“I would say that when I’m not around he is very good at taking care of it [dog]. He is able to recognize his needs. Anyway, the dog will tell him, when he hasn’t eaten, ... makes him understand that he hasn’t eaten. The dog, the communication, because it’s a non-verbal communication that has been established over time and I would say that it’s beautiful to see, myself I'm amazed at that.” (Companion dog, 26-09-2020, caregiver)

In Table 4, dyads with no dog have talked more about “*companionship and solicitude*”*.* Here are supporting quotes from the groupings:

“So, she’s there, hum... so she’s my protection, she’s my companion, she makes me be more responsible with things, like I have a job to do with her, which keeps my mind going somewhat.” (NSD, 07-05-2019 person with dementia)

“I mean, if my wife is not feeling well, she [dog] curls up and watches her. Or a couple weeks ago, she wasn’t well, and our dog - she went up to bed and stayed with her.” (Companion dog 17-05-2019, caregiver)

“It would give him company, he could go for a walk with the dog. He would have a small living being to take care of. I think that would stimulate him.” (No dog, 19-04-2021, caregiver)

#### Doing physical activity with the dog

Neuro service dogs are taught to promote physical activity in their owner. According to the World Health Organisation (2021), “Physical activity refers to all movement including during leisure time, for transport to get to and from places, or as part of a person’s work. Both moderate- and vigorous-intensity physical activity improve health. Popular ways to be active include walking, cycling, wheeling, sports, active recreation and play, and can be done at any level of skill and for enjoyment by everybody”. Walking the dog outside and taking care of it in the house increases the owner’s physical activity. This role was highly discussed in dyads with companion dogs and no dog (NSD 16%, companion dog 31%, no dog 37%). The sub-theme “Play with the dog” is supported in an equivalent way in all cases (Table 3). The “Walk with the dog” sub-theme is two times less discussed in the NSD group than in both other groups (Table 3). However, the quotes confirm that there is much more moderate- and vigorous-intensity physical activity reported by owners of NSD than walk the dog or play with the dog (light or sedentary physical activity).

“I do the routine with the kickboxing and the dog walking. I do that Monday to Thursday. I usually take our own dogs out and do a bunch of stuff on Fridays just walking and running at the park and go to the beach or whatever. And then, you and I usually take them on the weekend to do things. So, I’m very active. [...] I wear a Fitbit, all the time. I have reach 20,000 steps a day.” (NSD, 2020-09-30, person with early dementia)

“Well, when we’re in the city, he generally just goes in the backyard, we have a big backyard. So I… once in a while, we’ll walk along the river.” (Companion dog, 2019-05-18, caregiver)

“…we’re up and about moving around and playing with them and letting them out and walking around with them and stuff.” (Companion dog, 2020-01-27, caregiver)

“I) Okay, are you fairly physically active? D) Ah I try to be yeah. My Parkinson limits sometimes but… I) Okay, so do you think that the dog might help you with maintaining your physical activity? I) That is the main reason that we are getting it.” (No dog, 10-03-2019, person with dementia)

#### (Role in) sleep or wake up

Neuro service dogs are taught to assist with quality of sleep and waking up. It is recognized that 60–70% of people in the early stages of dementia present with disturbances of sleep and wakefulness (Wennberg et al., 2017), and that the reassuring presence of a dog could help them. By its presence, the dog helps to fall asleep or to go back to sleep, or even sometimes wake up its master for certain considerations. However, only one caregiver in the NSD group addressed this point, while nine caregivers did so for companion dogs. Very few caregivers or persons with dementia addressed the subthemes “Time it takes to fall asleep or time spent sleeping at night”, “Number and duration of awakenings during the night” and “Alarm clock”.

“So, intervening during a nightmare, ... C: Yes, oh yes, it's because he needs to get out of his nightmare and that's what I do. When he has nightmares, I get up and go calm him down.” (No dog, 07-06-2021, caregiver)

“When my husband has a nap, she (dog) is always right there and they are comforting each other. […]. Before my husband even gets up in the morning, she jumps up on the bed and she lick his face and wakes him up.” (NSD, 19-02-2019, caregiver)

“Oh yes! I definitely get a better sleep when the dog is lying beside me. It’s like a safer feeling.” (NSD, 30-09-2020, person with dementia)

“Yeah, he’d [dog] wait until I fell asleep and he’d go and cry on her side and she’d pick him up and put him on the bed. But, he’d know not to wake me up, but in the morning he’d be there, waiting for her to get up.” (Companion dog, 19-06-2019, caregiver)

### Advantages of having a dog

Table 4 shows seven benefits or advantages of having a dog. Also, it points out three specific themes where the research hypothesis H1 is supported in favor of the NSD group. **General feeling of security** is the theme mentioned by most participants in all cases (NSD 29%, companion dog 34%, no dog 36%) but only by 14 of the 23 participants in dyads with no dog. The related sub-themes are “Decreased anxiety or reduced stress” and “Developing friendship with the dog” (see Table 4). **Well-being and quality of life** is also widely supported in all three cases (NSD 31%, companion dog 28%, no dog 30%) but only reported by nine of the 23 participants in dyads with no dog. Similarly, the related sub-themes, “Have life meaning” and “Happiness and other positive feelings” are supported in a similar way regardless of the group (Table 4). **Dog monitors loved one’s sleep and napping** is the third most cited advantage by participants with a dog (NSD 21%, companion dog 13%, no dog 8%) but again only reported by four of the 23 participants in dyads with no dog (Table 4). Subthemes “Dog goes to bed with the loved one”, “Dog allows better sleep to the loved one” or “Dog allows better sleep to the caregiver” supported this advantage (Table 4). **Independence** is a theme that refers to the idea that having a dog in the home gives the caregiver more freedom to pursue other roles because the dog can take care of their loved one in their absence. In other words, the dog gives the caregiver independence. This is reported by 5/5 of the family caregivers of the NSD group, 20/28 of the companion dog group and 6/23 for the no dog group (27%). Related sub-themes are “Give the caregiver the opportunity to relax or some headspace to think about something else”, “Less anxiety for the caregivers” and “Allows the caregiver to leave home for a few hours” (Table 4). **Positive economic impact** of having as service dog at home is an advantage since it “Allows the caregiver to keep a job”, as cited by six caregivers in total (NSD 3%, companion dog 2%, no dog 0%). No group members supported the sub-theme, “Allows the caregiver to have a career”. Quotes from caregivers who have NSD in the home confirm H1 for these two themes, i.e., the presence of a NSD has more positive impact on the caregiver’s independence and on personal finances than the presence of a companion dog. Here are quotes supporting the first sub-theme.

“I’m about to take over a part time position there at the church. So, having the dog does enable me to go to work.” (NSD, 25-02-2019, caregiver).

“I: Is there an economic impact? C: Oh yes. Oh yes. Basically, when she gets home, I check on the camera [from my desk at work] if she’s in the room and the dogs are with her and I have no fear of her getting lost. So, that’s a big bonus. Because I know she’s active. And I can track her on my phone with two apps. So, when she does get somewhere she’s not supposed to, Sky does surveillance at home. I know where she is.” (Companion dog, 6-10-2020, caregiver)

**Positive focus on the dog** by the person with dementia is an advantage when, for example, taking care of the dog to keep him healthy rather than worrying about other things that might cause anxiety. This theme was seen as an advantage (NSD 2%, companion dog Group 5%, no dog 6%) mostly supported by 11 participants in dyads with companion dogs (Table 4). **Reminders** coming from the dog are seen as an advantage of having a NSD (NSD 2%, companion dog 0%, no dog 4%), but only reported by one participant with a NSD, one with a companion dog and three participants without dog. H1 is confirmed here, that presence of a NSD has more positive impact on the activation of the memory of the person with dementia (for doing things, routine) than the presence of a companion dog.

### Disadvantages, inconveniences, and obstacles of having a dog

Table 4 shows the 10 inconveniences reported in the three cases. Also, it points out one specific theme where the research hypothesis H2 is supported in favor of the NSD group. The highest percentage of quotes in the category of disadvantages goes for the dyads who do not have a dog, for the **Maintenance** supported with six sub-themes shown in Table 4 (NSD 29%; Companion dog 27%; no dog 53%). The theme **Fears and disinterests surrounding having a dog** is also an important inconvenient for dyads with no dog (NSD 7%; Companion dog 7%; no dog 14%). Here are some quotes that support this.

“The only issue I had was, being that he was not a service dog or trained to be a service dog, I was scared that he would trip the wife. Being that she had dementia, she’d forget that he’s around and he’d get in between her legs or something, and trip her.” (Companion dog, 30-09-2019, caregiver)

“Well, the disadvantage is when you have mobility issues like I have now, that you can easily trip over them ... If they’re not trained just to stay out the way like a service dog will perhaps be. They’re not trained to stay out of the way, so they don’t […] the cats and the dog will do that sometimes, they will try to run real fast in front of me when I’m going back to the bedroom and cut me off. I mean they can just cut you right off and yeah because they’re that fast. Yeah and so I had to watch that because they’ll do that like late at night when you’re not watching and so that can be a danger. I have problems with stairs. So that's an issue.” (Companion dog, 30-04-2020, caregiver).

**Reaction of employees or clients** if negative can be a real inconvenient; it is supported by four sub-themes (NSD 19%; companion dog 11%; no dog 8%). Even though it rarely happens when the dog is a service dog, a clinic clerk or staff’s rejection of a service dog can be profoundly inconvenient. The subtheme “Ban on dogs in shops” is definitively the obstacle encountered with companion dogs. This confirms H2 where the presence of a NSD results in the person with dementia accessing more indoor and outdoor public sites than with a companion dog. Following are quotes from owners of NSD and companion dogs referring to “Ban on dogs in shops” and “Bad people’s reaction to dogs.”

“When she gets the first service dog, she went to the Dollar store, and they say that she couldn’t have the dog in there. […]. I wrote an email to the company, and they ended up having a staff meeting the following day, because they were just being ridiculous”. (NSD, 30-09-2020, caregiver)

“And yesterday, M. was at a doctor’s appointment, and we believe the doctor just really doesn’t like Teagan being in there, and when that doctor came to the room he just stuck… opened the door, stuck his head in the room and said something, and Teagan responded with a bark. And then, hum… So the doctor asked… said for her not to take Teagan there anymore. And we understand this, we know that… we think it was excessive on the doctor’s part but at the same time Teagan is not [supposed] to bark in public. (NSD, 25-01-2019, caregiver).

“In USA, we don’t go anywhere inside with the dogs usually. In the coffee shops, the dogs have to stay outside”. (Companion dog, 28-02-2020, caregiver).

“Even going to the hardware store, they look at us with big eyes and say “go put your dog in the car”. The only mall where they accept dogs is Place de la Cité. So, again, it’s not a nice mall to walk around in ... I would like to be able to take them everywhere, but it seems that they are afraid that they will do their business. I always have bags and try to be careful, but maybe some people don’t pick up after themselves like I do. Maybe that’s why there are a lot of stores and then shopping malls where they don’t want to see it”. (Companion dog, 27-01-2020, caregiver translated)

**Dog’s health problem or bad behavior** is an inconvenient and it is supported by three sub-themes in Table 4 (NSD 19%; companion dog 17%; no dog 9%). This theme more discussed in dyads who have a dog. Here are quote related to this theme:

“Most trained service dogs, well they have times when they are ill like a bladder infection or something. You just never know. Most service dogs can hold on for a long time.” (NSD, 19-02-2019).

“C: At the gym. Uh that’s another incident that happened, […] it’s a very tense situation, because M. was exercising on a machine, and this other lady walked around to access a machine nearby, and that startled Teagan. And so she barked. And then M. got anxiety and said, “I think I better take her home”. And the person that’s there said it was probably a good idea. But we … I think the person that was there, the staff person, was thinking more of M. being anxious about it… than to the dog barking. Nobody had… as I heard M., it seemed like nobody had a negative response to the dog barking, except M”. M: “Yes… This was the first time, I mean I’ve been going down there for three months now, and this was the first time she barked and they said this was so unusual.” (NSD, 25-01-2019, caregiver and person with dementia).

**Inappropriate focus on the dog** is a disadvantage when the person with dementia lacks discipline or authority for the dog’s well-being. This happens when it is a pet dog (NSD 0%; companion dogs 6%; no dog 1%).

“If I can tell you quickly, a small part of the daily life is that my mother, because she has dementia, she forgets things and she feeds the dogs. Every time, I tell her that she should not feed the dogs, because there are things that are toxic for the dogs. What happens is that I discipline the dogs, ... because they bark for nothing or ... they jump. I discipline them and when I baffle them, they go to her.” (Companion dog, 24-09-2019, caregiver translated).

Common to all three cases, the theme of **Dealing with the absence of the dog** is often mentioned as a disadvantage, concerning death of the dog or placement in residential care. Some people do not want to have a dog because of the grief that comes when it passes away (NSD 7%, companion dogs 18%, no dog 4%).

“We were never able to get another. As soon as you see a dog, you immediately think of the other and it was such a good dog and it’s hard to see if you could have one that would be like him there.” (No dog, 15-11-2021, caregiver)

“I know that, let’s say I’m in a support group and there’s someone in there, that her spouse has been in residence for many years. Her son has a dog. ... when he goes to see his dad, he brings the dog. ... it’s allowed. So, if I ever had the dog with me probably I would bring it when I go to see my spouse if they allow it.” (Companion dog, 26-09-2020, caregiver translated)

“The placement, I have thought of that and I have wondered how that would work, and it scares me to think of it, and so I find since we don’t know hundred percent how the future is going to fold out. I’m trying my best to cope by that mindfulness and live one day at the time. When I start to think about what will become of Yukon and R., if and when that time comes, I am so attached to her but I don’t know.” (NSD, 19-02-2019).

The theme **Dog’s need for break time** (for meal, sleeping, toileting site) was mentioned as an inconvenience by eight participants (NSD 1%, companion dog 2%, no dog: 0%). The need to access canine toileting facilities during travel was particularly challenging.

“Our dog is… she had the mistake (of defecating) three times so far in public, we did everything we could to make sure that she was relieved, but you know at an **airport**, uh she (the dog) went in the comfort room, and she just had a big old pile right in the middle of a walkway. There, we circled it with our luggage and ourselves, I ran off and M. (the person with dementia) picked it up, and then I came out with towels and soap and cleaned it up. Unfortunately, it’s going to happen once to them all… [… ]. The airport, they had the facilities, but she (the dog) wouldn’t use them. […]. The lady who had supervision of the facility actually dropped in while we were there, and she said many dogs who are very responsible, just will not go in those kind of artificial environments”. (NSD 25-01-2019, caregiver).

Common to all three cases, **People’s reaction in the street** was encountered as an inconvenience by only three participants (NSD 2%, companion dog 1%, no dog 0%). However, the size or a less popular dog breed may scare people in public.

“C: Because of the size of the big ones, most of the time people back off and they kind of get nervous and scared. People that like animals will make comments, like “oh how pretty”, “that's nice” or “they’re good to get it”, they’ll make positive comments, and people that are fearful of animals they are negative. They more often will shy away or cross the street. I: Okay. And the fact that the two of your dogs are Pitbull’s, are there some people afraid of that because of the breed? C: I think that's the main reason why they get afraid of it. People have a negative tone to their breed”. (Companion dog, 09-07-2020, caregiver)

The **Dog acquisition and training** (time, cost) appears to be an inconvenient but not a matter in dyads with a companion dog (NSD 5%, companion dogs 1%, no dog 6%). One dog school may charge an expensive price (no waiting list) and another school may absorb the cost with donations (long waiting list).

“So I kept searching and I found the company, I found a couple. One in Vancouver and then this one in Winnipeg and we went with them. Now to have a dog trained through a company. It is pricey. We paid 25,000 CAN$ dollars, which is a lot.” (NSD, 19-02-2019, caregiver)

“Well our daughter… well it took a year and a half to two years before the dog was trained” (NSD, 10-04-2019, caregiver)

“The total cost, I think, was around 15,000 to 16,000 US $ dollars, with transportation, room and board, and those kinds of things. The dog itself was 14, 500 US $ dollars under contract, and that’s about… that’s a little over half of what the actual costs are.” NSD, 25-01-2019, caregiver?

The **Extra care and cost required for the dog** is discussed in the same proportions in the three cases (NSD 7%, companion dogs 8%, no dog: 7%). It was about surgery, medication, medical monitoring.

## Discussion

### About the objectives

The first research objective is met because we examined the impact of canine assistance on engagement, meaning of life, socialisation, sense of direction, physical activity, sleep and other participation activities for both the person with dementia and his/her caregiver. In this regard, our study documented six specific roles and usages of canine assistance. The Socialisation and Help with a sense of direction roles are unique to NSD, as well as Other unexpected usages. Engagement-and-meaning of life and Physical activity roles were more addressed in dyads with companion dogs and without dog. Actually, it seems the NSD is trained to do particular physical activities with the person with dementia, but not necessarily the companion dog. The role of Sleep or wake up appears to be a less predominant role for both the NSD or the companion dog, compared to the other roles. Moreover, our content analysis reveals additional impacts of canine assistance in terms of seven advantages and 10 inconvenient. Previous literature on service dogs with other populations (motor impairments, PTSD) is different in terms of roles and usage and advantages, but similar regarding disadvantages (Lessard et al., 2018; Vincent et al., 2016). This is the first study that has examined the impact of canine assistance on both the person with dementia and their caregiver living in their home. Previous literature reports only animal assisted intervention in nursing homes and for persons with moderate to severe dementia (Briones et al., 2021; Kårefjärd & Nordgren, 2019; Lai et al., 2019; Majić et al., 2013; Olsen et al., 2016a, 2016b, 2019; Parra et al., 2022; Wesenberg et al., 2019).

The second research objective is met because we documented the informal acceptance of canine assistance in public places. The companion dog is considered the friend of the person with dementia, as it walks with them, sometimes reminds them to eat, and contributes to their socialization (EODAF, 2022). In the disadvantages, we can see that a NSD is sometimes not welcomed in some public places even if they are certified for public access. Companion dogs are not authorised in public places, but most of the time, they are accepted by people on the street.

Our qualitative results fully support the mechanism of “Motivation - responsibility of caring” included in final CMO configurations for the Dementia Dog Project (Ritchie et al., 2021) through the first role and usage of the NSD (Engagement-and-meaning-of life: Obligations, responsibilities, or valued occupation towards the dog). This also corroborates the themes from the three studies with phenomenological hermeneutic approaches reported in Marks & McVilly (2020). The mechanism “Human–animal bond” addressed in Ritchie et al. (2021) is also supported by the same role but for another subtheme (Companionship and solicitude). Our results fully support the six outcomes through the roles associated with the NSD (Socialization, Physical activity with the dog) and the advantages (General feeling of security, Independence, Well-being and quality of life, Positive focus on the dog). Our results about Inconvenients (Maintenance, Acquisition and training, and Dealing with the absence of the dog support) partially support the results of Marks & McVilly (2020).

### About the hypotheses

Our general hypothesis had captured the differences of conceptualisation of having a NSD or a companion dog or no dog at all. The category of *Roles and uses* category is better supported in the case of the NSD, which makes sense given the actual presence of an NSD and that the owner of the dog is the person with dementia. The category of *Benefits* is better supported in the case of companion dogs, which makes sense given that the owner is more often the caregiver, they have had a pet dog for several years and would often like their loved one to have an NSD where they see even more benefits. Finally, the category of *Inconvenients* is better supported in the case without a dog, as caregivers give examples where cognitive function, safety and risk of falls are more considerable in their home and older persons with older partner than the other cases.

Our first hypothesis H1 (the presence of a NSD has more positive impacts on both the person with dementia and their caregiver compared to the presence of a companion dog) is supported by three roles and three benefits as shown in Tables 3 and 4; in this sense H1 is confirmed both by the NSD and companion dogs groups but differently. The NSD encourages moderate and vigorous-intensity physical activity in the routine of the person with dementia (as it is trained for public access) while the companion dog encourages more quiet walks and play in the house. Additionally, the NSD can guide the person with dementia in their daily activities outside the home, and brings them back safely. Similarities between these groups have also been observed for the other roles, especially if the owner of the companion dog is really the person with dementia. Both NSD and companion dogs help enhance engagement by the person with dementia in the completion of daily routines and that this gives meaning to their lives. They both can promote quality of the sleep and attends to awakenings.

Our second hypothesis H2 (the presence of a NSD results in the person with dementia accessing more indoor and outdoor public sites than with a companion dog) particularly concerns one role and one inconvenient as mentioned in Tables 3 and 5; in this sense H2 is confirmed both by the case of NSD and companion dogs but in a different way. The NSD facilitates the socialization of the person with dementia, several examples have been reported of the person being approached by others in a store, a restaurant, an airport, a gym, a church and a clinic, which goes beyond the socialization during walks with the dog.

Our third hypothesis H3 (dyads of persons with dementia and caregivers with a NSD or a companion dog are more informally socially engaged more frequently than those with no dog) is supported by one role as pointed out in Table 3; in this way H3 is confirmed but differently across the three cases. People who love dogs will come up and talk about the dog, and the persons with dementia can talk about their dog. The dog often becomes a topic of conversation that starts the socialization process. The results of our three hypotheses in favor of a NSD support five of the six outcomes provided by the final CMO configurations for the Dementia Dog Project (Ritchie et al., 2021). Here are outcomes that correspond to H1 (emotional support for the couple, reduction in caregiver stress and worry, and care and support for the person with dementia, their partner and the dog) and to H2 and H3 (increase in activity, maintenance of routine).

This study also documented situations where canine assistance is contraindicated. The dyads without dogs provided reasons that a dog is not desirable in their home. The question of maintenance or daily care of the dog was the most predominant reason. Many caregivers in this group mentioned that their loved one often fell as the disease progressed. They indicated the fear of losing balance and falling when the dog is in the way. The research team has also observed that caregivers without dog in our study have a higher score on the Caregiver burden scale than in the other two groups, and the person with dementia without dog have more advanced dementia and more of them have Parkinson. In Parkinson’s disease, walking becomes less automatic (Abou-Sharkh & Mayo, 2022). The person with the disease must make a greater effort and concentrate to maintain an efficient and safe gait. In our study, one of the five people who acquired a NSD had Parkinson’s (39 years old) with mild dementia. She did not report that her dog kept her from losing her balance or that she fell. This suggests that the stage of disease (mild) combined with a NSD may be protective for safe and effective walking. In our study, four of these five individuals had mild dementia and were younger than in the other two groups.

**Figure 2.**
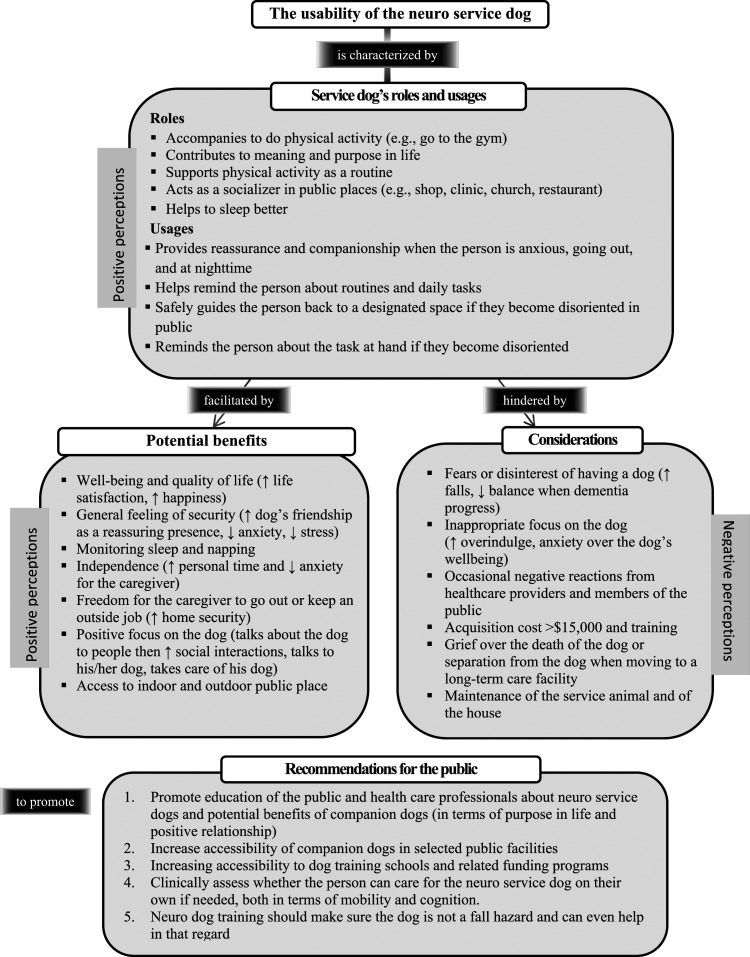
Usability of the neuro service dog for the caregiver living with a person with dementia: a proposed conceptual framework. Legend: ↑ (increase or improve), ↓ (decrease or reduce).

Based on this discussion and on the results obtained, Figure 2 proposes a conceptual framework of the usability of the NSD for caregivers living with a person with dementia. It suggests that the usability of the NSD involves five roles and four usages. Its usability is facilitated by seven potential benefits and six considerations are important. Five recommendations are provided for the public aimed at increasing facilitators and decreasing obstacles to improve the NSD usability, as well as companion dogs.

### Methodological strengths and limitations

The internal validity of the study is not fully met, since there were only five dyads in the NSD group as the training school closed in 2020 due to the Covid pandemic. We believe that if we had been able to recruit closer to 10 people, we could have claimed theoretical saturation because we would have maximised internal diversification of the sample (Krefting, 1991), for the Case 1─ dyads with NSD. This is also true for the Case 3 ─ dyads with no dog ─ where we have to adapt the interview guide for people “who don’t want a dog” when we were no longer able to reach “people on the dog school waiting list”. Maybe with better internal diversification of those cases, the “unexpected usages of a dog” would have been less miscellaneous and would have resulted in other unique roles to a neuro service dog. With respect to the external validity of the study, the transferability of the data to a similar context is possible since we have provided exhaustive sociodemographic information for the three cases, for the persons with dementia, the caregiver and the dog, and transcripts with context. The fact of having proposed three visual hypotheses in a diagram and of having reported them in the three tables of results facilitates the transfer of data to corroborate the qualitative results from one study to another (Chigbu, 2019). Participants were recruited from different provinces of Canada (except four of the five NSD) and lived in a variety of home settings. Cases 1 and 2 have different size and type of dog (NSD vs. companion). We believe that the data can be generalized to comparable geographic and meteorological contexts. Regarding the external reliability of the study, we feel we achieved optimal consistency since we explained in detail the procedure for developing themes and sub-themes in the methodology section, at least for the roles and usage of NSD. Finally, concerning the internal reliability of the study, doing parallel coding of the interviews three times with two other evaluators ensured better confirmatory potential of the study results (Krefting, 1991; Parmar et al., 2014).

Further research is needed to better document the evolution of NSD roles and usages over time, as well as the benefits and drawbacks. The results of the present study were cross-sectional, and data was collected between three and fourteen months following the acquisition of a NSD.

## Conclusion

This is a first study recruiting 112 participants on phone, person with dementia living with their caregiver. Their voice confirmed qualitatively that the presence of a neuro service dog has a greater positive impact on persons with dementia living at home with their caregiver, than those with an untrained companion dog. By impact, we mean that the NSD contributes to a greater extent to physical activity, sense of direction and better sleep and wake up of the person with dementia. Results also demonstrate that the presence of a NSD allows the person with dementia to have more opportunities for social engagement compared to the presence of a companion dog due to the possibility of access to inside and outside public facilities. This study also highlights the fact that caregivers living with their loved one who has dementia may not benefit from canine assistance when dog maintenance is considered a chore; when the loved one’s condition is progressing (especially if they are at high risk of falling) and when they have difficulty bending or walking normally. It would be too difficult for the loved one to manage the dog’s daily routine and it would be an additional burden for the caregiver. Longitudinal research would be welcome to document the full value of NSD roles and usages.
